# Exposure to Environmental Organic Mercury and Impairments in Human Fertility

**Published:** 2019

**Authors:** Geir Bjørklund, Jan Aaseth, Maryam Dadar, Monica Butnariu, Salvatore Chirumbolo

**Affiliations:** 1- Council for Nutritional and Environmental Medicine, Mo i Rana, Norway; 2- Research Department, Innlandet Hospital Trust, Brumunddal, Norway; 3- Inland Norway University of Applied Sciences, Elverum, Norway; 4- Razi Vaccine and Serum Research Institute, Agricultural Research, Education and Extension Organization (AREEO), Karaj, Iran; 5- Banat’s University of Agricultural Sciences and Veterinary Medicine “King Michael I of Romania” from Timisoara, Timisoara, Romania; 6- CONEM Romania Biotechnology and Environmental Sciences Group, Banat’s University of Agricultural Sciences and Veterinary Medicine “King Michael I of Romania” from Timisoara, Timisoara, Romania; 7- Department of Neurological and Movement Sciences, University of Verona, Verona, Italy

It is widely known that mercury (Hg) is highly toxic to humans. Environmental Hg pollution still represents a huge health concern worldwide (1). Human industrial activities have led to raised levels of Hg in the air, soil, and fresh and sea waters, and to bioaccumulation along the food chain. Humans easily absorb Hg through fish consumption. Also, it is a major toxicant because it has adverse effects on human reproductive health (2). Mercury, particularly in its organic forms, methyl-Hg and ethyl-Hg, is toxic even when individuals are exposed to relatively low Hg levels (3). Toxic effects of Hg can easily be observed in the nervous, digestive, and immune systems, in addition to lungs, kidneys, skin, and eyes, as reported by the World Health Organization (4). Many of these effects are mediated by the host’s immune response via its limited detoxification and metal excretion capabilities. Genetic polymorphism of scavenging Hg-complexed proteins are factors that can induce stressor effects, which also depend on the capability of enzymatic detoxification systems, aside from the dose of the toxicant (5).

The evidence that organic Hg easily crosses the placental barrier and reaches the fetus represents another great concern. Thereby, Hg is a major cause of neural developmental defects including delayed postnatal development (6). Both elemental Hg^0^ and methyl-Hg easily cross the placenta, and it should be emphasized that the developing fetal brain is the most sensitive organ. However, the more recent debate about the toxicity of dietary Hg in fetal or prenatal toxicology (7) has revealed controversies about Hg effects on fetal development, particularly because it is difficult to establish a reliable Hg-pharmacokinetics in adult women undergoing pregnancy. The pharmacokinetics and threshold of plasma Hg-acceptable levels in pregnancy are still matters of debate, and updated evidence-based official guidelines are generally lacking (8).

Recent reports together with the previous information from the Minimata and Iraq disasters have reignited this debate (9). Mercury exposure may explain cases of idiopathic infertility in males as well as in females (10). Moreover, it affects chromosomes or embryonic cell DNA, presumably by its interference with one-carbon transfer since a methionine carrier transports it across membranes (11). Recent evidence has reported that Hg inhibits the energy metabolism of spermatozoa, thus preventing their normal functioning (12). Methyl-Hg causes reproductive organ damage in women (13).

Furthermore, menstrual disorders, sterility, and spontaneous abortion following abnormal Hg toxicology have been reported (14). Spontaneous abortion can occur when the pregnant woman is exposed to organic Hg or other toxic agents (15). Many spontaneous abortions do not have a known cause, but reports indicate that Hg exposure might be a triggering factor in several cases (16). Paternal exposure to Hg, and to other industrial chemicals, and pesticides are also presumed to play a role 17). Studies on a Chinese population revealed that neural tube defects were associated with exposure to methyl-Hg in pregnancy and higher placental deposition of the compound (18). Based on the current knowledge, great concerns have been raised regarding Romanian districts with remarkably high Hg pollution (19).

Regarding pre-and postnatal risks of Hg exposure, it is relevant that the concentrations of Hg in the nervous system and other target organs of children may be higher than in the mothers, in part due to the small weight of a newborn child associated with a high rate of gastrointestinal absorption and low renal excretion, implying that hazardous Hg exposure may continue after birth (20).

Taken together, the comments presented here illustrate that the relationship between Hg toxicology and fertility has still been scarcely addressed in the literature, despite previous lessons from Minimata and some few reports in recent years. Apparently, Hg exposure may have a considerable impact on multiple stages of reproduction, from before conception to the maturation of organs and endocrine systems and further to the healthy development of the child. Therefore, a continued systematic investigation and assessment of the current state of knowledge about congenital and developmental anomalies with possible associations to environmental pollutants are of particular importance.
